# Fiber-type composition and 3D capillary analysis of the human splenius capitis muscle

**DOI:** 10.17305/bb.2024.10787

**Published:** 2024-09-10

**Authors:** Nataša Pollak, Chiedozie Kenneth Ugwoke, Nejc Umek, Armin Alibegović, Jiří Janáček, Barbora Radochová, Erika Cvetko

**Affiliations:** 1 Institute of Anatomy, Faculty of Medicine, University of Ljubljana, Ljubljana, Slovenia; 2 Institute of Forensic Medicine, Faculty of Medicine, University of Ljubljana, Ljubljana, Slovenia; 3 Laboratory of Biomathematics, Institute of Physiology of the Czech Academy of Sciences, Prague, Czech Republic

**Keywords:** Skeletal muscle, splenius capitis muscle, vastus lateralis muscle, capillary network, myosin heavy chain (MyHC) isoforms, 3D image analysis, human

## Abstract

Despite the significance of neck muscles in musculoskeletal disorders, their microscopic anatomy remains poorly characterized. This study examined the splenius capitis muscle, focusing on its fiber-type composition, fiber size, and capillary network characteristics. For comparison and validation, the vastus lateralis muscle was also analyzed. Muscle samples from 13 young male subjects (mean age ± SD: 35.7 ± 8.6 years) were collected within 24-h post-mortem during autopsy. Myosin heavy chain (MyHC) isoform expression was characterized immunohistochemically in 10 µm sections, while the capillary network architecture was assessed in 100 µm sections. Immunofluorescence staining, confocal microscopy, and 3D image analysis were employed to quantify capillary tortuosity, anisotropy, branch density (Br dens), and the length of capillaries per muscle volume (LV), per muscle fiber length (LL), per fiber surface area (LS), and per fiber volume (LVf). Compared to the vastus lateralis muscle, the splenius capitis muscle had a higher percentage of type 1 fibers (51.2% vs 39.7%), fewer type 2a fibers (16.2% vs 31.4%), and smaller fiber diameters (35.5–40.9 µm vs 47–56.1 µm). It also displayed lower Br dens (*P* ═ 0.0069), higher anisotropy (*P* ═ 0.0004), and lower LL (*P* < 0.0001) but higher LVf (*P* ═ 0.0486). In the splenius capitis muscle, body mass index (BMI) negatively correlated with LV (*P* ═ 0.0155), LS (*P* ═ 0.0091), LVf (*P* ═ 0.0137), and anisotropy (*P* ═ 0.0425), and positively correlated with tortuosity (*P* ═ 0.0473), indicating a reduction in the capillary network. In the vastus lateralis muscle, only LV (*P* ═ 0.0161) decreased with high BMI. This study characterized the fiber-type composition, fiber size, and 3D capillary network of the splenius capitis muscle, establishing a baseline for investigations into pathological muscle alterations.

## Introduction

The cervical part of the vertebral column contains a complex network of over 16 pairs of muscles that connect the skull, cervical and thoracic vertebrae, and the shoulder girdle. These muscles play a crucial role in stabilizing head posture, controlling head movements, and generating multidirectional forces [1]. One of these muscles, the splenius capitis, is a multifunctional muscle located deep to the trapezius and superficial to the semispinalis capitis. It originates from the spinous processes of the lower four cervical and upper three thoracic vertebrae and inserts into the superior nuchal line, the nuchal plane of the occipital bone, and the mastoid process of the temporal bone [2]. The splenius capitis muscle is responsible for head and neck extension when activated bilaterally, and lateral flexion and rotation toward the same side when activated unilaterally. It also helps maintain head and neck posture during movements like prone extension, free extension, rotated chin-ups, and head protrusion [3]. The muscle is innervated by the posterior ramus of the spinal nerves C2 and C3 [2, 4], with its primary blood supply coming from the occipital artery and additional contributions from the deep cervical artery and the thyrocervical trunk [2].

Neck muscles, including the splenius capitis, are increasingly being recognized for their significance in spinal disorders [5]. Studies on cervical muscles in individuals with neck pain—primarily using ultrasound and MRI—[6] have linked neck pain to altered muscle function, atrophy, and fat infiltration [5, 7, 8]. The splenius capitis muscle has also been associated with chronic tension-type headaches [9, 10], and its impaired postural function is implicated in individuals with forward head and rounded shoulder posture, often linked to prolonged desktop device use [11].

Despite the prevalence of cervical spine pathologies and related muscle impairments, the fiber-type composition and capillary network characteristics of neck muscles, including the splenius capitis, remain poorly understood in healthy individuals. Fiber types determine muscle contractile properties [12], while capillary density is critical for nutrient supply [13]. We hypothesize that the splenius capitis muscle, given its role in postural control and movement, exhibits a complex pattern of myosin heavy chain (MyHC) isoform expression and capillary network structure, reflecting both its physiological and pathological functions. Previous studies on splenius muscle fiber types have focused on patients with cervical dysfunction or did not differentiate between splenius capitis and cervicis muscles [14, 15]. There is a lack of data on fiber-type composition, size, and capillary network characteristics in healthy individuals.

To address this gap, this study aimed to investigate the fiber-type composition, size, and 3D capillary network characteristics of the splenius capitis muscle in healthy individuals. To validate the findings and confirm the robustness of the 3D methodology, which has not previously been used to study neck muscle capillary networks, the more extensively studied vastus lateralis muscle was included as a comparative control. The data from this study provide a valuable baseline for understanding the microanatomy of the splenius capitis muscle, aiding in the assessment of pathological muscle alterations.

## Materials and methods

### Muscle specimens

Samples of the splenius capitis and vastus lateralis muscles were collected within 24-h post-mortem from 13 male individuals aged 23–48 years, who had died suddenly due to traumatic accidents or sudden cardiac death. The average age of the subjects was 35.7 ± 8.6 years, with a mean body mass index (BMI) of 26.5 ± 4.4 kg/m^2^. No pathological alterations were observed in the muscle tissue sections. Muscle samples were collected during standard autopsies performed at the Institute of Forensic Medicine, University of Ljubljana. Subjects were included if they had no history of physical disorders, medical conditions, or treatments known to affect muscle phenotype. Information regarding lifestyle and physical activity levels was not available from medical records. Subjects with traumatic damage to the target muscles were excluded. Due to the limited number of autopsies performed on women aged 18–50 years, females were excluded from the study. The splenius capitis muscle samples (∼2 × 2 cm) were taken from the mid-portion of the right muscle, near the C4 vertebral level. Similarly, vastus lateralis muscle samples of comparable size were obtained from the superficial middle section of the muscle. Following collection, the samples were rapidly frozen in liquid nitrogen and stored at –80 ^∘^C until histological analysis.

### Immunohistochemistry

#### MyHC expression analysis

Serial 10 µm thick transverse sections of muscle samples were prepared using a Leica CM 1950 cryo-microtome (Leica Microsystems, Germany). Monoclonal antibodies diluted in phosphate-buffered saline (PBS) were applied to these sections to assess the expression of MyHC isoforms. BA-D5 antibodies, reactive to ß/slow MyHC-1 in humans and rats [16, 17], were applied at a 1:100 dilution. SC-71 antibodies, reactive to MyHC-2a and MyHC-2x/d in humans [18] and dogs [19], were also diluted at 1:100. Additionally, 6H1 antibodies reactive to MyHC-2x/d in humans [20] were applied at a 1:50 dilution. The Polymer Detection System (Novolink, Leica Biosystems, UK) was used to detect the MyHC-2x/d isoform. Other MyHC isoforms were revealed using the indirect immunoperoxidase method, with secondary antibody P0260 (Dako, Denmark) as per previously described protocols [21, 22]. Nonspecific binding was minimized by pre-incubating sections in a blocking solution of normal rabbit serum (dilution 1:40) and 0.5% bovine serum albumin in PBS. Specificity was confirmed by the absence of immunoreactivity in control sections without primary antibodies.

### Capillary analysis

Thick transverse sections (100 µm) were preserved in 4 ^∘^C PBST and fixed using 7% formaldehyde and 0.1% glutaraldehyde in PBST. Antigen retrieval was performed by incubating the sections at 37 ^∘^C for 5 min in 0.5-M Tris buffer (pH 8.0) with EDTA and 0.2% proteinase K. To label the basal lamina, sections were incubated overnight at a 1:200 dilution with a rabbit polyclonal antibody against collagen IV (Abcam, UK), followed by rinsing in PBST and application of a secondary antibody with Alexa Fluor 546 (1:500; Invitrogen, USA). Capillaries and endothelial cells were visualized using fluorescein-labeled Griffonia (Bandeiraea) simplicifolia lectin I (1:300; Vector Laboratories, USA) and F8/86 (1:1000; Dako, Denmark). The secondary antibody Alexa Fluor 488 (1:500; Invitrogen, USA) was applied to visualize F8/86 binding sites. Sections were embedded in ProLong™ Gold Antifade Mountant (Molecular Probes, USA). Specificity was confirmed by the absence of immunoreactivity in control sections without primary antibodies.

### Image acquisition and analysis

#### MyHC expression analysis

High-resolution images (5440 × 3648 pixels) of the serial sections stained with MyHC isoform-specific antibodies were captured using a Nikon Eclipse 80i microscope (Nikon Corporation, Japan) equipped with a 20× objective and a KERN ODC 841 camera (KERN & SOHN GmbH, Germany). Microscope VIS Pro KERN OXM 902 software (KERN & SOHN GmbH, Germany) was used for image acquisition. To ensure a representative sample, at least three fields of view were randomly selected within each muscle, capturing a minimum of 100 fibers per muscle (total area: 829.87 × 103 µm^2^). The Ellipse image analysis program (ViDiTo, Košice Slovakia) was used to delineate each muscle fiber. Muscle fiber classification and analysis were performed using the software by Karen et al. (2009), which categorized fibers as type 1, type 2a, type 2x, and hybrid fibers co-expressing MyHC isoforms (type 1/2a and type 2a/2x). The average diameter and numerical proportions for each fiber type were estimated. Image acquisition and analysis were performed by a single operator (M.S.), blinded to group assignments.

### Capillary analysis

Random sampling was used to select a minimum of five fields of view per muscle for capillary analysis. Each field contained 20–60 fibers, ensuring that at least 100 fibers were analyzed per muscle. Each field was 387.5 µm × 387.5 µm, providing an overall area of 750.78 × 103 µm^2^. A Leica STELLARIS confocal microscope (Leica Microsystems, Germany) equipped with an HC PL APO CS2 40×/1.1 water immersion objective was used for imaging. Stacks of 8-bit images were captured at 1-µm intervals with a resolution of 512 × 512 pixels and a pixel size of 0.756 µm × 0.756 µm, using the LAS X 4.6.0.27096 software. Excitation wavelengths of 488 and 570 nm (White Light Laser, pulsed at 78 MHz and tunable from 440–790 nm) were employed. To prevent crosstalk between channels, emission signals were filtered using an acousto-optical beam splitter, prism-based dispersion, and mirrors, and images from the two channels were collected sequentially.

**Figure 1.**
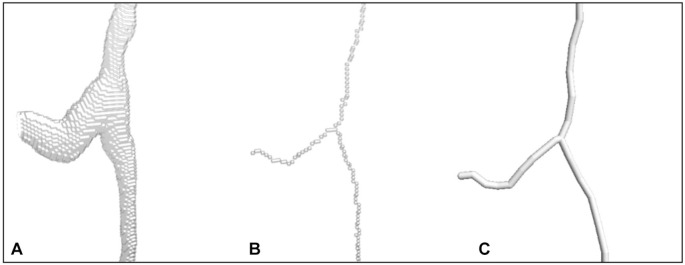
**3D Analysis of the capillary network structure.** (A) 3D binary image of capillary branching; (B) 3D skeleton obtained by 6-pass Palágyi algorithm; (C) Geometric model—3D graph composed of linear segments.

The Ellipse program 2.081 (ViDiTo, Slovakia) was used for further analysis. To account for shrinkage, image stacks were subjected to axial calibration, as described by Janáček et al. [23]. Binary images were skeletonized using the Palágyi algorithm and manually refined with a haptic device (Figure 1). Parameters, such as fiber diameter, surface area, and volume were computed based on muscle fiber outlines observed at four levels within the image stack [24].

Various capillary network measurements were made, including capillary length per fiber, line segment lengths within a 10 µm fiber neighborhood were summed, and these measurements were expressed relative to muscle fiber length and volume, following the method described by Janáček et al. [25]. Additionally, capillary length parameters were computed in relation to muscle fiber surface and fiber volume. The mean capillary length was calculated as 2/3 of the total capillary length divided by the number of branching points [24, 26]. The equation: used was: 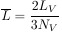, where 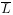 is the average capillary length, *L_V_* is the capillary length per unit volume, and *N_V_* is the number of branching points per unit volume.

Branching density (Br dens) was calculated, and capillary tortuosity was measured by dividing the sum of the exterior angles (in radians) between consecutive line segments by the total capillary length. The anisotropy index, indicating the capillary network’s isotropy or anisotropy, was computed using eigenvalues obtained from the structural tensors of the line segment directions [26]. All analyses were conducted in a single-blind manner, with the operator unaware of group assignments.

### Ethical statement

The study protocol was approved by the National Medical Ethics Committee of the Republic of Slovenia (Permit No.: 0120-536/2019/4).

### Statistical analysis

All statistical analyses and graphical representations were performed using GraphPad Prism 10 (GraphPad Software, San Diego, CA, USA). The Shapiro–Wilk test was used to assess the normal distribution of continuous data. Repeated-measures two-way ANOVA with Šidák post hoc tests was used to compare numerical proportions and diameters between muscles and fiber types. Paired *t*-tests were used to compare capillary parameters—such as capillary length per muscle volume (LV), Br dens, tortuosity, anisotropy, capillary length per muscle fiber length (LL), capillary length per fiber surface (LS), and capillary length per fiber volume (LVf)—between the splenius capitis and vastus lateralis muscles. Spearman’s correlation coefficient was used to test linear correlations between variables. Statistical significance was set at *P* < 0.05.

## Results

### Muscle fiber type and size

**Figure 2.**
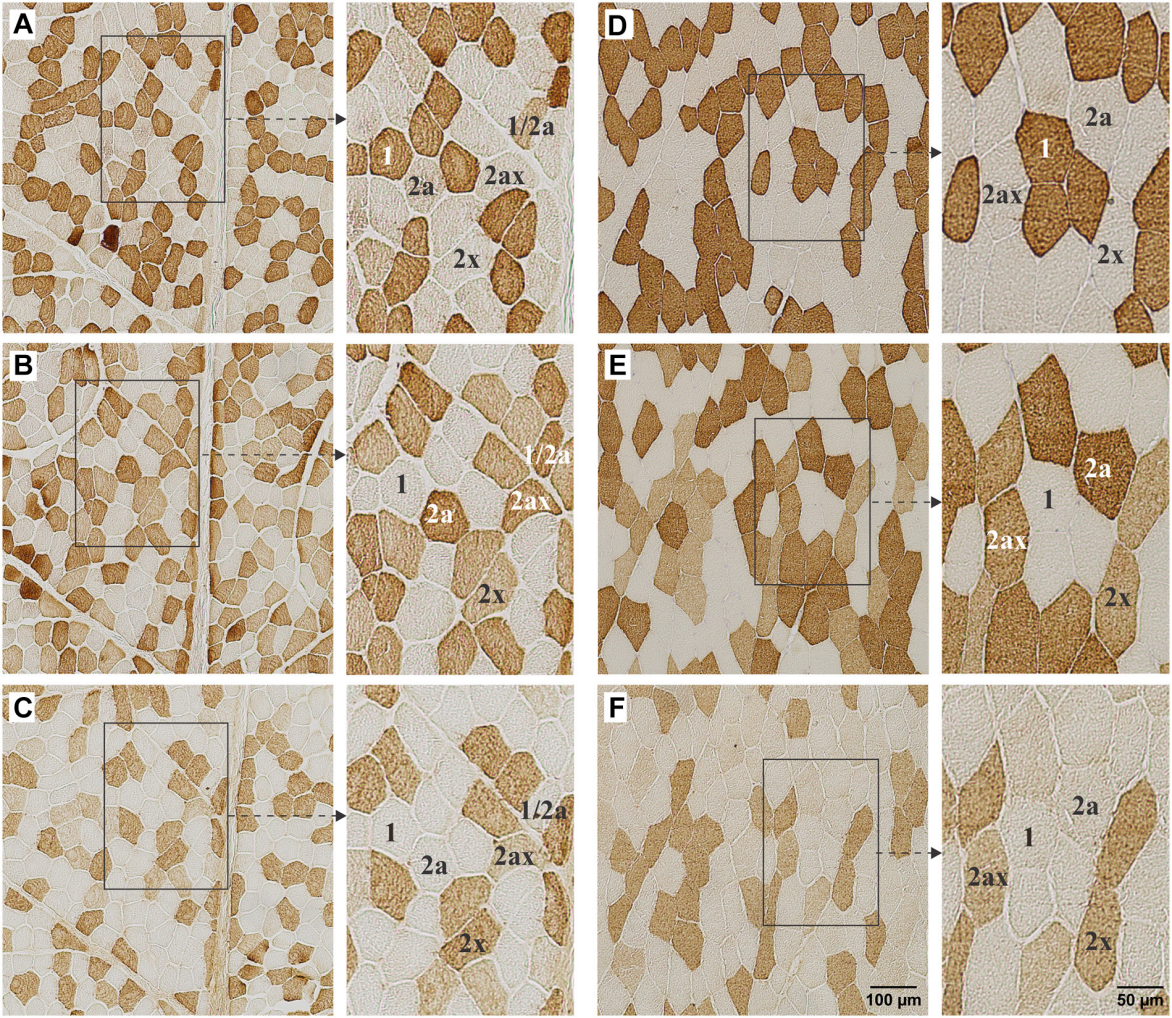
**MyHC isoform expression in splenius capitis and vastus lateralis muscles of a 24-year-old male.** Expression of MyHC isoforms 1, 2a, and 2x, coexpression of 2x and 2a, and coexpression of 1 and 2a in a 24-year-old male in successive cross-sections of splenius capitis muscle (A–C) and vastus lateralis muscle (D–F). The adjacent image to the right (bar scale 50 µm) of each photomicrograph (bar scale 100 µm) shows the magnification of the portion of the original image indicated by a box and arrow. Fibers expressing different MyHC isoforms are labeled. MyHC: Myosin heavy chain.

**Figure 3.**
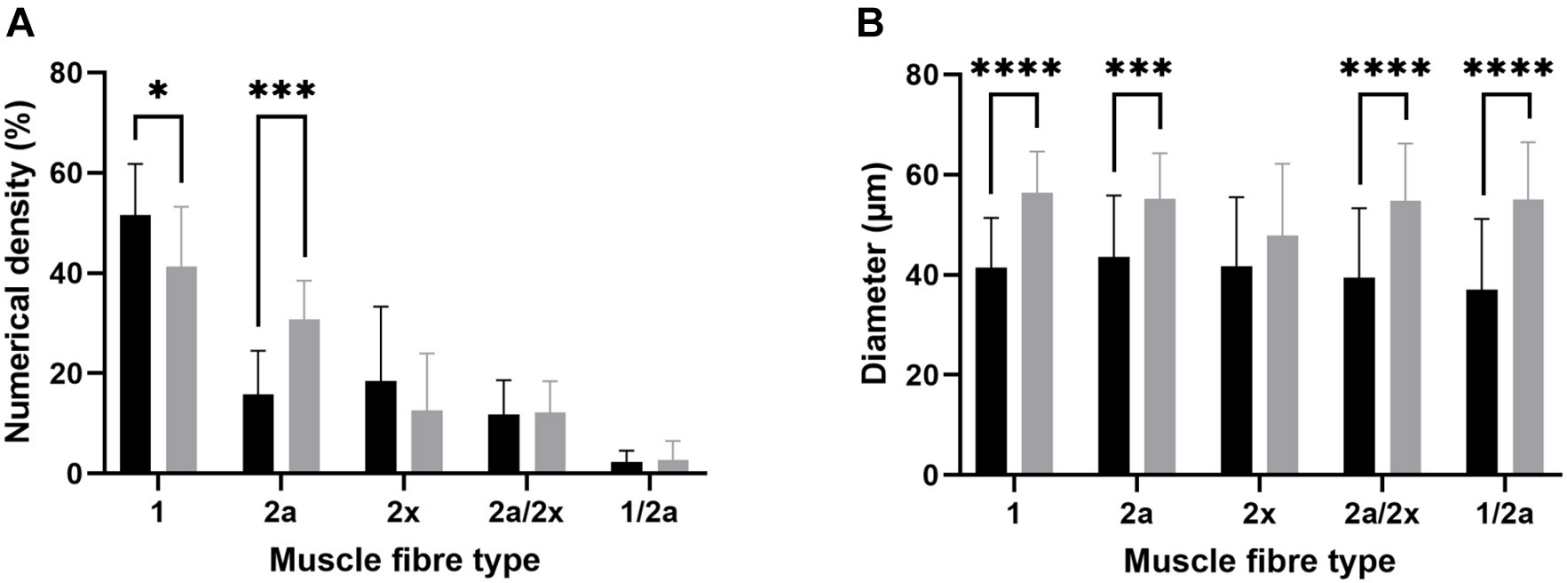
**Numerical density and diameter of muscle fibers expressing particular MyHC isoform in the splenius (black) and vastus lateralis (gray) muscle of 13 young males.** Statistical significance between the muscles is indicated by the asterisks above the bars: **** denotes *P* < 0.0001, ***denotes *P* < 0.001, and * denotes *P* < 0.05. MyHC: Myosin heavy chain.

Both the splenius capitis and vastus lateralis muscles displayed a mosaic distribution pattern, characterized by the random arrangement of fiber types within the muscle cross-section. This pattern includes both slow and fast fiber types, resulting in a mixed fiber composition with no clear compartmentalization (Figure 2). The distribution of fiber types is shown in Figure 3A. Hybrid fibers (type 1/2a) were absent in 25% of the samples. The splenius capitis muscle exhibited a significantly higher proportion of type 1 fibers (51.2% ± 9.8%) compared to the vastus lateralis muscle (39.8% ± 15.0%, *P* ═ 0.0023), while the vastus lateralis muscle had a significantly greater proportion of type 2a fibers (31.4% ± 7.7% vs 16.3% ± 8.4%, *P* ═ 0.0006). The percentage of type 2× fibers was slightly higher in the splenius capitis muscle (17.4% ± 8.9% vs 14.1% ± 7.4%), but the difference was not statistically significant. Both muscles showed similar proportions of hybrid type 2a/2x fibers (13.0% ± 7.9% in the splenius capitis vs 12.9% ± 6.4% in the vastus lateralis) and hybrid type 1/2a fibers (2.6% ± 2.4% in the splenius capitis vs 1.8% ± 1.1% in the vastus lateralis).

Muscle fiber size estimates, based on diameter, are presented in Figure 3B. The splenius capitis muscle fibers were approximately 30% smaller in diameter compared to those in the vastus lateralis muscle. Type 1 fibers in the splenius capitis muscle measured 37.5 ± 8.6 µm, while in the vastus lateralis muscle, they were 56.2 ± 7.9 µm (*P* < 0.0001). Type 2a fibers had diameters of 38.1 ± 10.0 µm in the splenius capitis vs 55.1 ± 8.7 µm in the vastus lateralis (*P* ═ 0.0001). Type 2x fibers were 41.7 ± 13.6 µm in the splenius capitis compared to 47.1 ± 13.8 µm in the vastus lateralis, but the difference was not statistically significant (*P* ═ 0.1028). Hybrid fibers (2a/2x and 1/2a) in the splenius capitis muscle measured 35.2 ± 9.6 µm and 38.9 ± 8.0 µm, respectively, compared to 54.2 ± 11.0 µm and 53.3 ± 12.2 µm in the vastus lateralis muscle (*P* < 0.0001 and *P* ═ 0.0001, respectively). Given that many studies assess fiber dimensions using cross-sectional area (CSA) rather than diameter, CSA was also calculated to enable comparisons with other studies. In the splenius capitis muscle, the mean CSA values were: type 1 (3849 ± 1031 µm^2^), type 2a (3880 ± 1219 µm^2^), type 2x (3072 ± 1453 µm^2^), type 2a/2x (3548 ± 1342 µm^2^), and type 1/2a (3460 ± 1095 µm^2^). For the vastus lateralis muscle, the corresponding values were: type 1 (7663 ± 2214 µm^2^), type 2a (8387 ± 2438 µm^2^), type 2x (6247 ± 1824 µm^2^), type 2a/2x (7314 ± 2061 µm^2^), and type 1/2a (7017 ± 1985 µm^2^).

There were no statistically significant relationships between age or BMI and the fiber-type proportions or diameters for either the splenius capitis or vastus lateralis muscles.

### 3D analysis of the capillary network

Significantly higher Br dens (1.44 ± 0.46 µm^−3^ × 10^−6^ vs 0.92 ± 0.24 µm^−3^ × 10^−6^) was observed in the vastus lateralis muscle (*P* ═ 0.0069), while greater anisotropy (2.35 ± 0.44 vs 1.61 ± 0.14) was noted in the splenius capitis muscle (*P* ═ 0.0004). However, no significant differences were observed between the two muscles when evaluating tortuosity (33.8 ± 11.7 in the splenius capitis vs 40.9 ± 8.6 in the vastus lateralis; *P* ═ 0.3110) or LV (408.7 ± 70.5 in the splenius capitis vs 409.2 ± 70.6 in the vastus lateralis; *P* ═ 0.6258).

**Figure 4.**
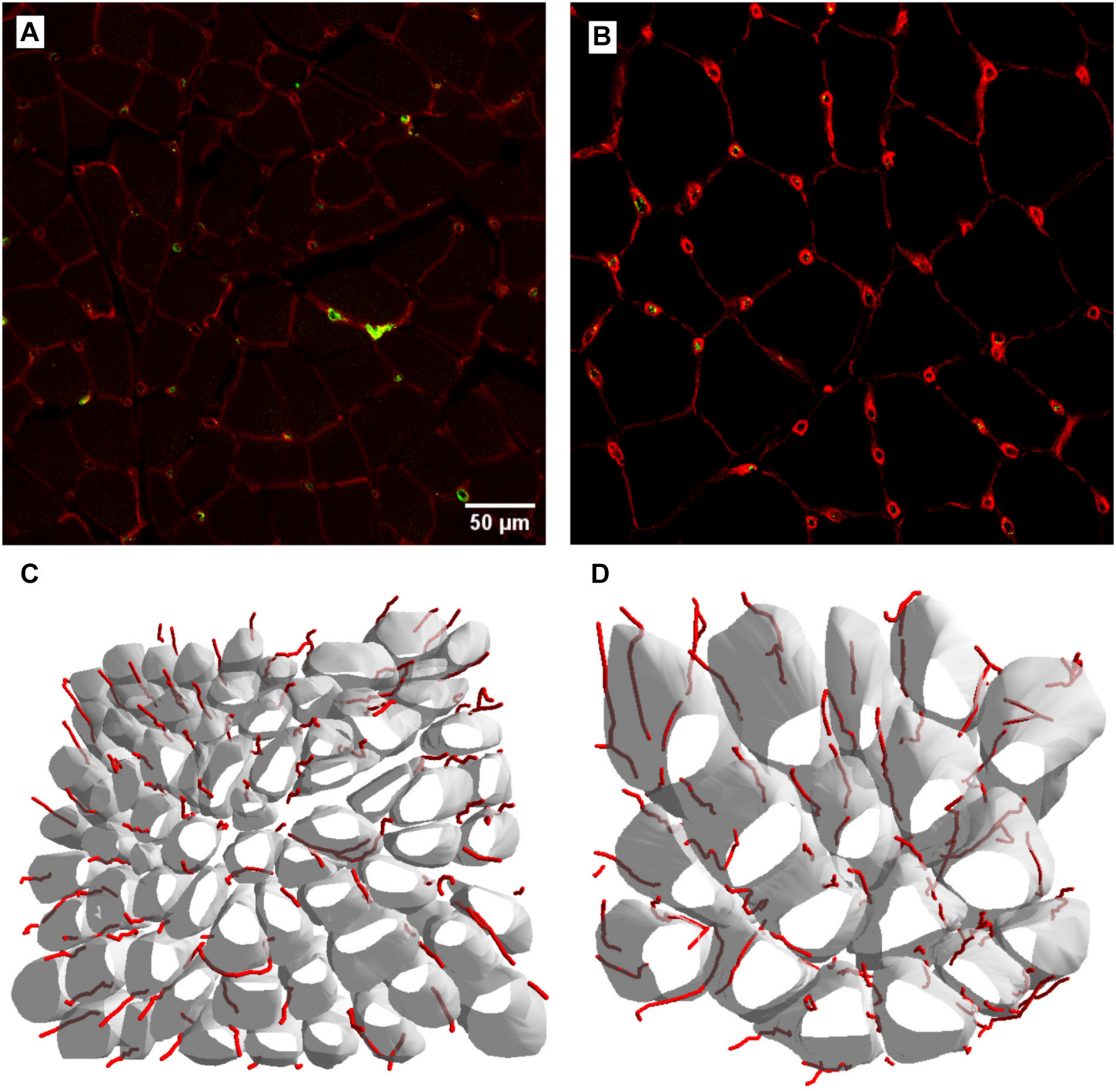
**Capillaries and muscle fibers in splenius capitis (A and C) and vastus lateralis (B and D) muscles.** Images A and B represent merged images of the green and red channels of triple immunofluorescence staining for capillaries (in yellow-green) and muscle fiber outlines (in red). Both images represent a selected slice from the stack, providing a visual depiction of the spatial distribution within the 3D reconstruction. Images C and D represent a 3D rendering of capillaries and muscle fibers in the splenius capitis (C) and vastus lateralis (D) muscles. Bar scale ═ 50 µm.

Additionally, the splenius capitis muscle exhibited lower LL (2.1 ± 0.3 vs 3.9 ± 0.8; *P* < 0.0001) and LS (132 ± 17 µm^−1^ × 10^−4^ vs 165 ± 32 µm^−1^ × 10^−4^; *P* ═ 0.0038), but a higher Lvf (13.1 ± 3.7 µm^−2^ × 10^−4^ vs 10.9 ± 2.6 µm^−2^ × 10^−4^; *P* ═ 0.0486). The capillary network density, as estimated by LL and LS, was smaller in the splenius capitis muscle compared to the vastus lateralis. Results are summarized in Table 1, and representative images of immunohistochemical staining and 3D renderings of capillaries and muscle fibers from both muscles are shown in Figure 4.

**Table 1:** Capillary properties of muscle fibers in splenius capitis and vastus lateralis muscle

	**Splenius capitis**	**Vastus lateralis**	***P* value**
LVm [µm^−2^] × 10^−6^	408.7 ± 70.5	409.2 ± 70.6	0.6258
LL	2.1 ± 0.3	3.9 ± 0.8	**<0.0001**
LSf [µm^−1^] × 10^−4^	132 ± 17	165 ± 32	**0.0038**
LVf [µm^−2^] × 10^−4^	13.1 ± 3.7	10.9 ± 2.6	**0.0486**
Tortuosity [rad µm^−1^] × 10^−3^	33.8 ± 11.7	40.9 ± 8.6	0.3110
Anisotropy	2.35 ± 0.44	1.61 ± 0.14	**0.0004**
Br dens [µm^−3^] × 10^−6^	0.92 ± 0.24	1.44 ± 0.46	**0.0069**

Capillary network characteristics were estimated by the length of capillaries per volume of muscle tissue (LVm) ([µm^−2^] × 10^−6^), length of capillaries per length of muscle fibers (LL), length of capillaries per fiber surface (LSf [µm^−1^] ×10^−4^), length of capillaries per fiber volume (LVf [µm^−2^]) ×10^−4^, tortuosity [rad µm^−1^] ×10^−3^, anisotropy, number of branching per muscle volume (Br dens [µm^−3^]) ×10^−6^. Data are mean ± standard deviation, and figures in **bold** print indicate statistically significant *P* values.

Significant linear correlations were also noted between BMI and LS (ρ ═ –0.690, *P* ═ 0.0091), LVf (ρ ═ –0.662, *P* ═ 0.0137), LV (ρ ═ –0.653, *P* ═ 0.0155), tortuosity (ρ ═ 0.558, *P* ═ 0.0473), and anisotropy (ρ ═ –0.569, *P* ═ 0.0425) for the splenius capitis muscle. In the vastus lateralis muscle, only the linear correlation between BMI and LV (ρ ═ –0.662, *P* ═ 0.0161) was significant.

## Discussion

### Fiber-type composition

The human splenius capitis and vastus lateralis muscles display different proportions of the three primary muscle fiber types (type 1, type 2a, and type 2x) as well as hybrid fibers that express two or more MyHC isoforms. The splenius capitis muscle is a mixed muscle with nearly equal proportions of type 1 and type 2 fibers (Figures 2 and 3A). Previous research on the splenius capitis muscle is limited. Uhlig et al. [14] analyzed biopsy samples from patients with post-traumatic neck instability and identified the proportions of type I, type IIA, type IIB, and transitional type IIC fibers using myosin ATPase histochemistry. In their study, type IIB fibers correspond to type 2× fibers, as identified in this study by immunohistochemistry [27]. The present study’s findings on type 1 and type 2× fiber proportions align with those of Uhlig et al. (Table 2). However, our study found a lower proportion of type 2a fibers (16.2 ± 8.4% vs 26.6 ± 14.2%) and a higher proportion of hybrid fibers (2a/2x: 12.9 ± 6.4%; 1/2a: 2.6 ± 2.4%) compared to their report of 0.7% transitional fibers. While the role of hybrid fibers remains unclear, they could represent transitional states, gradual MyHC isoform replacements, or stable entities with combined fiber-type properties that enhance muscle performance and adaptability [28–31]. The relatively higher proportion of hybrid fibers in this study might reflect the use of more sensitive methodologies rather than pathological changes.

**Table 2:** Comparison of results of fiber-type composition and fiber size of the splenius capitis muscle with findings of previous studies

**Authors/ Year**	**Type of sample/number of subjects/sex**	**Age (years) Mean ± SD**	**Medical condition**	**Muscle**	**Method**	**Percentage of muscle fiber types (%)**					**Diameter of muscle fiber types (µm ± SD), cross-sectional area (µm^2^ ± SD)**				
						**Type 1**	**Type 2**		**Hybrid**		**Type 1**	**Type 2**		**Hybrid**	
							**2a**	**2x**	**2a/2x**	**1/2a**		**2a**	**2x**	**2a/2x**	**1/2a**
Uhlig et al., (1995) [14]	Biopsy 15 M Biopsy 7F	44.5 ± 14.6 34.6 **±** 11.3	Post-traumatic instability	Splenius capitis	ATPase histochemistry	54.9 ± 13.3 55.4 ± 8.9	26.6 ± 14.2 20.6 ± 7.0 (IIA)	17.8 ± 14.6 23.4 ± 8.3 (Type IIB)	No data	No data 0.7 ± 0.8 (Type IIC)	No data	No data	No data	No data	No data
Vikne et al., (2012) [15]	Autopsy 12 (11 M + 1 F)	45 ± 15.8	Presumably healthy	Splenius	Ab to MyHC-1 and MyHC 2 subtypes	51.6 ± 11.5	17.2 ± 7.6	12.7 ± 12.3	18.3 ± 7.6	No data	CSA 1971 ± 546	CSA 1845 ± 706	No data	CSA 1645 ± 660	No data

Ab: Antibodies; D: Diameter; CSA: Cross-sectional area.

Vikne et al. [15] used immunohistochemistry to determine the MyHC fiber-type composition in the splenius muscle of healthy individuals but did not specify whether the analysis was conducted on the capitis or cervicis region. Their findings on type 1 and type 2a fibers are consistent with the results of the present study, although slight differences were observed in the proportions of type 2x and hybrid 2a/2x fibers. Detailed numerical data are provided in Table 2.

The results of the present study on the vastus lateralis muscle are consistent with the findings of Vikne et al. [32], who conducted a systematic review. They reported pooled weighted means of 46.9% ± 10.8 for type 1 fibers, 39.4% ± 9.5 for type 2a fibers, and 12.7% ± 7.3 for type 2× fibers in young, healthy individuals, using ATPase histochemistry or immunohistochemical staining. This consistency supports the validity of the analysis techniques used and highlights the distinctive characteristics of each muscle based on its function. While the vastus lateralis muscle, with its fiber distribution, is well-suited to its weight-bearing role, the fiber-type composition of the splenius capitis muscle may reflect adaptations for both rapid contractions and resistance to fatigue, particularly during extended periods of maintaining head posture. Previous studies have shown that muscles involved in postural functions are typically dominated by type 1 fibers, while muscles with predominantly phasic activities exhibit a higher prevalence of type 2 fibers [33].

### Fiber size

The mean diameter of splenius capitis muscle fibers ranged from 35.5 to 40.9 µm (Figure 3B), with no significant difference between type 1 and type 2 fibers. Vikne et al. [15] provided only partial data on the CSA of splenius muscle fibers (Table 2). When comparing the present study’s results for the splenius capitis muscle with reports on other neck muscles, it was noted that the splenius capitis muscle has smaller type 1 and type 2 fibers than the sternocleidomastoid but similar type 1 CSA to the suboccipital muscles (Table 3) [21, 34].

**Table 3:** Comparison of fiber-type composition and fiber size of the neck muscles

**Authors/Year**	**Type of sample**	**Sex**	**Age (years) Mean ± SD or age (range)**	**Muscles**	**Histochemical method**	**Percentage of muscle fiber types (%)**			**Diameter of muscle fibers types (µm ± SD),** **cross-sectional area (µm^2^ ± SD) or** **proportional area occupied by type I fibers** **(% ± SD)**		
						**Type 1**	**Type 2**		**Type 1**	**Type 2**	
							**2a**	**2x**		**2a**	**2x**
Cornwall et al., 2016; Yamauchi et al., 2017 [34], [54]	6 autopsy samples 3 autopsy samples	M M	79.2 ± 6.9 (53–97)	Rectus capitis posterior major	Ab to MyHC-1 and MyHC-2, but not type 2 subtypes	60.1 ± 8.9			1828 ± 43 µm^2^	1654 ± 77 µm^2^	1484 ± 152 µm^2^
				Rectus capitis posterior minor		58.8 ± 9.5			2098 ± 54 µm^2^	1056 ± 57 µm^2^	1435 ± 144 µm^2^
				Obliquus capitis superior		61.3 ± 12.3			2462 ± 77 µm^2^	1047 ± 48 µm^2^	2069 ± 98 µm^2^
				Obliquus capitis inferior		69.2 ± 10.5			1771 ± 41 µm^2^	1711 ± 57 µm^2^	1252 ± 81 µm^2^
Lindman et al., 1990 [36]	5 autopsy samples	M	29 (16–38)	Trapezius descending part 1	ATPase histochemistry	67 ± 7	21 ± 6	11 ± 7	3957 ± 787 µm^2^	4429 ± 1312 µm^2^	4070 ± 1185 µm^2^
				Descending part 2		66 ± 11	29 ± 7	5 ± 4	3732 ± 804 µm^2^	4291 ± 1417 µm^2^	3782 ± 1578 µm^2^
				Descending part 3		69 ± 4	28 ± 4	3 ± 2	3870 ± 400 µm^2^	5440 ± 511 µm^2^	5216 ± 932 µm^2^
Cornwall & Kennedy, 2015 [55]	4 embalmed cadavers	M	87.25 ± 7.93 (79–95)	Scalenus anterior	Ab to MyHC-1 and MyHC-2, but not type 2 subtypes	71.9 ± 15.0			83.7% ± 6.0%		
				Scalenus medius		64.0 ± 13.7			79.7% ± 10.2%		
				Scalenus posterior		52.3 ± 13.6			76.0% ± 8.48%		
				Longus capitis		48.5 ± 11.0			61.4% ± 9.1%		
				Longus colli		50.0 ± 11.1			64.1% ± 8.0%		
Cvetko et al., 2012 [21]	15 autopsy samples	M	30.3 ± 6.3 (18–40)	Sternocleidomastoideus	Ab to MyHC-1 and MyHC 2 subtypes	31.5 1/2a:4.1 1/2a2x:1.1	29.7	4.3 2a2x: 26.8	46.3 ± 2.3 µm	46.9 ± 3.3 µm	2a2x: 46.3 ± 2.4 µm

Ab: Antibodies; MyHC: Myosin heavy chain.

The mean diameter of vastus lateralis muscle fibers in this study was 53.2 µm, with no significant difference observed between the diameter of type 1 and type 2 fibers. Although previous research has demonstrated larger fiber sizes in type 2a fibers of the vastus lateralis muscle in adult males, no difference was observed between the diameters of type 1 and type 2 fibers in adult females or children [35]. The distinct functional roles of limb and neck muscles likely account for the development of smaller, type 1 muscle fibers in the neck, which are better suited for activities requiring endurance and precise motor control [36]. Conversely, the larger fibers in the vastus lateralis reflect its load-bearing role. Other factors, such as physiological CSA [37], muscle fiber length [38], capillary density [39], mitochondrial density [40], and neuromuscular activation patterns [41], also contribute significantly to muscle function and adaptation. Further research is necessary to fully understand these variables and their interactions. Notably, while oxidative capacity is generally inversely related to fiber size, the classification of fibers based on MyHC isoform expression does not always align with their oxidative capacity. Smaller fibers can exhibit higher oxidative enzyme activity and mitochondrial density, regardless of fiber type [42].

### 3D analysis of the capillary network

This study is the first to examine the capillary network characteristics of the splenius capitis muscle or any cervical muscle. The 3D methodology used here has been previously applied by Janáček et al. [26] to describe the capillary architecture of the vastus lateralis muscle. Although their sample size was limited (*n* ═ 5) and composed of older males (mean age 58, range 32–87), the observations of vastus lateralis capillary parameters align with the findings in the present study, which further validates the use of this analytical method in a younger cohort. Notably, a significant correlation was found between BMI and several capillarization indices in the splenius capitis muscle (LV, anisotropy, tortuosity, LS, LVf). BMI accounted for roughly 40% of the variability in capillarization, indicating that a higher BMI is associated with a decrease in capillarization. This trend was less pronounced in the vastus lateralis muscle, which may be due to a training effect conferred by its weight-bearing role. Previous research has suggested that weight-bearing muscles are more resistant to structural changes associated with increased BMI, particularly in terms of fiber-type shifts [43]. This raises questions about the suitability of these muscles for studying obesity and related metabolic phenotypes.

The splenius capitis muscle showed a higher capillary length per fiber volume but not per muscle volume when compared to the vastus lateralis muscle, likely due to the smaller diameter of splenius capitis fibers [44]. This smaller diameter may favor oxidative metabolism, as shorter diffusion distances between muscle fibers and surrounding capillaries can enhance oxygen uptake [35]. While smaller fibers exhibit higher maximal oxygen consumption, larger fibers may encounter limitations in oxygen uptake under optimal supply conditions [42]. Although the splenius capitis muscle has a higher capillary length per fiber volume, the vastus lateralis muscle has a greater Br dens, resulting in a higher capillary LS. The lower capillary branch density, higher anisotropy, and longer capillaries per fiber length in the splenius capitis muscle may represent a specialized adaptation, while the vastus lateralis muscle, with its role in weight-bearing and lower limb movements, requires a denser capillary network to meet its functional demands.

Historically, capillarization in skeletal muscles has been evaluated using two-dimensional (2D) analyses of tissue cross-sections [42, 45, 46], which can significantly underestimate capillary length, especially in muscles with complex, non-parallel fiber arrangements [47]. The present study employed a well-validated 3D method previously used to characterize skeletal muscle capillarization [24, 26, 47–49]. This 3D approach provides a more accurate and comprehensive view of capillary networks, making it particularly useful for identifying subtle changes in the microvasculature.

### Study limitations

This study has several limitations. First, the use of post-mortem muscle specimens presents challenges related to sample integrity and potential post-mortem alterations. While biopsy samples typically offer better tissue preservation, their small size limits the ability to conduct comprehensive analyses, including fiber phenotype characterization and 3D capillary analysis. Additionally, while commonly used antibodies were effective in thin sections, they proved challenging in thick sections of human muscle due to inconsistent staining and poor tissue penetration. For this reason, alternative dual labeling with lectin and the F8 antibody was employed, offering better tissue penetration than CD31.

Second, the small sample size and exclusion of females may limit the generalizability of the findings. Caution should be exercised when drawing broad conclusions [50, 51]. The cohort, considered “healthy” based on the absence of known premortem diseases, had a mean BMI in the overweight range. Despite a careful review of medical records, the study lacked detailed lifestyle and dietary data that could provide additional context. Furthermore, fat infiltration in muscle tissues was not analyzed. Including fat infiltration in future studies could help establish baseline fat content and allow comparisons to pathological changes. Although fat infiltration is a significant feature in neck muscle pathology related to neck pain and whiplash [6, 52], it may not be consistently present in healthy individuals with chronic neck pain [53]. Future research should include measures of fat infiltration, particularly in healthy subjects, to improve understanding of muscle health and pathology in both males and females.

Future research should also aim to address these limitations by increasing sample sizes to include both males and females, incorporating detailed lifestyle data, and exploring the relationship between mitochondrial phenotypes, capillarization, and other parameters. Additionally, potential variations across sex, age, and disease states should be explored.

## Conclusion

In conclusion, the present study provides the first comprehensive characterization of the fiber-type composition, fiber size, and 3D capillary network architecture of the human splenius capitis muscle using advanced immunohistochemistry, confocal laser microscopy, and 3D image analysis. The observed differences, notably the higher proportion of type 1 fibers and smaller fiber diameters in the splenius capitis muscle compared to the vastus lateralis, underscore the distinct functional specializations of cervical muscles in postural control and movement. The 3D analysis of capillary networks revealed a significant correlation between capillarization parameters and BMI in splenius capitis muscle, providing new insights into how vascular architecture adapts to the muscle’s metabolic demands. Despite similar capillary LV between the splenius capitis and vastus lateralis muscles, the splenius capitis muscle exhibited increased anisotropy, lower branch density, and reduced capillary length relative to muscle fiber length and volume, suggesting a more specialized capillary arrangement suited to its functional role. Overall, the findings provide important insights on the complex fiber-type expression and capillary network properties that influence the physiological and pathological behavior of the splenius capitis muscle.

## Data Availability

The datasets used and analyzed during the present study are available from the corresponding author upon reasonable request.
